# Effects of marital status on survival of retroperitoneal liposarcomas stratified by age and sex: A population‐based study

**DOI:** 10.1002/cam4.4962

**Published:** 2022-06-27

**Authors:** Yiding Li, Guiling Wu, Yujie Zhang, Wanli Yang, Xiaoqian Wang, Lili Duan, Liaoran Niu, Junfeng Chen, Wei Zhou, Jinqiang Liu, Daiming Fan, Liu Hong

**Affiliations:** ^1^ State Key Laboratory of Cancer Biology and National Clinical Research Center for Digestive Diseases, Xijing Hospital of Digestive Diseases Fourth Military Medical University Xi'an China; ^2^ School of Aerospace Medicine Fourth Military Medical University Xi'an China; ^3^ Department of Histology and Embryology, School of Basic Medicine Xi'an Medical University Xi'an China

**Keywords:** marital status, retroperitoneal liposarcomas, socio‐psychosocial factors, surveillance, epidemiology, and end results (SEER) database, survival

## Abstract

**Background:**

Previous studies have shown that marital status is associated with survival in patients with a variety of cancer types, including lung cancer, prostate cancer, and bladder cancer. However, to date, the impact of marital status on the survival of patients with retroperitoneal liposarcomas (RPLs) has not been established.

**Methods:**

A total of 1211 eligible patients diagnosed with RPLs were identified in the Surveillance, Epidemiology, and End Results (SEER) database. The relationships between marital status and survival in patients with RPLs were assessed. Patients were stratified by age to determine whether an association exists between marital status and age. We also probed the association between marital status and survival in males and females.

**Results:**

Our findings suggest that divorced, separated, or widowed patients have more advanced cancer stages, and more of these patients do not undergo surgery. Meanwhile, divorced, separated, or widowed patients have worse survival outcomes than married patients (overall survival (OS): HR = 1.66 (95% CI, 1.12, 2.46)); cancer‐specific survival (CSS): HR = 1.90 (95% CI, 1.13, 3.19)). OS does not differ between single patients and married patients (HR = 1.21 [95% CI, 0.81, 1.81]) or CSS (HR = 1.36 [95% CI, 0.80, 2.29]). In addition, these results demonstrate that being divorced, separated, or widowed can play a significant detrimental role in mortality in older and female patients.

**Conclusion:**

Married patients have earlier disease stages at diagnosis and better survival outcomes than divorced, separated, or widowed patients with RPLs. In addition, this effect is especially pronounced in older people and females.

## Graphical abstract

Effects of marital status on survival of patients with retroperitoneal liposarcomas.

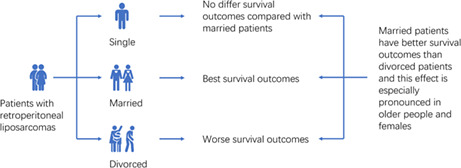

## 1 INTRODUCTION

Liposarcoma is a rare malignant tumor of the soft tissues and accounts for approximately 20% of all adult sarcomas.^1^, ^2^, ^3^ Liposarcoma arises within adipose tissue, occurring most commonly in the extremities, followed by the retroperitoneum.^2^, ^4^, ^5^, ^6^ However, the prognosis of retroperitoneal liposarcomas (RPLs) is worse than that of extremity liposarcomas (ELs).^6^ RPLs consists of several subtypes, with each subtype having different clinical, biological, and imaging characteristics. Consequently, the diversity of RPLs presents serious problems for studying RPL pathogenesis, hindering the development of novel therapies. For most patients with RPLs, early diagnosis and complete surgical resection are effective methods to improve survival. However, RPLs are unresponsive to most chemotherapeutic agents, and the use of radiation therapy is significantly restricted due to tissue toxicity, preventing the delivery of an adequate dose.^7^, ^8^, ^9^, ^10^ In addition, recent studies have demonstrated that novel therapeutic strategies, including targeted drugs, are effective for treating RPLs.^11^, ^12^ Despite recent advances in surgery, radiotherapy, and novel treatment strategies, the overall 5‐year survival rate of patients with RPLs remains at approximately 50%.^13^, ^14^

In previous studies, a large number of clinical and histopathological features and therapeutic strategies have been investigated to predict and improve the prognosis of RPLs.^15^, ^16^, ^17^, ^18^, ^19^, ^20^ With the increased understanding of human health and the consequent advancements in healthcare, the role of socio‐psychosocial factors in cancer is receiving increased attention. Several clinical studies have shown that socio‐psychosocial factors are important prognostic factors in a variety of tumor types in both adults and adolescents,^21^, ^22^, ^23^, ^24^ and marital status is one of the most important socio‐psychosocial factors.^25^, ^26^, ^27^ Recent studies have focused on marital status and cancer, and the results suggest that married patients have significantly better survival than unmarried patients.^27^, ^28^, ^29^ However, to date, there have been few studies on the effect of marital status among patients with RPLs.

In this analysis, we aim to analyze and explore the effects of marital status on RPLs using a relatively large number of cases obtained from the Surveillance, Epidemiology, and End Results (SEER) database. Patients were stratified by age to determine whether there was an association between marital status and age. We also probed the association between marital status and survival in males and females.

## 2 METHODS

### 2.1 Data source and population selection

The data source in the study was the SEER database provided by the National Cancer Institute's SEER*Stat software version 8.3.9.2 (https://seer.cancer.gov/data‐software/). Patients were considered eligible for participation in this study if they met all of the following criteria: (1). available data in the SEER 18 Regs Custom Data (with additional treatment fields), November 2018 Sub (1975–2016 varying) database; (2). an International Classification of Diseases for Oncology (ICD‐O) site code of C48.0 (retroperitoneum); (3). an ICD‐O‐3 pathological type of liposarcoma (8850–8858); and (4). year of diagnosis between 2004 and 2015. The exclusion criteria were as follows^1^: Age younger than 18 years and^2^ incomplete clinical and demographic characteristics, treatment, and follow‐up information.

Study variables included demographic information (age, sex, race, marital status), clinicopathological characteristics (histological grade [grade], morphology/pathological classification, primary site, SEER stage, AJCC stage, AJCC T stage, AJCC N stage, AJCC M stage), primary treatment modality (surgery), survival time, vital status and cause‐specific death classification at last follow‐up. X‐tile software (version 3.6.1) was used to analyze the optimal cut‐off point (65 years old) for age.^30^ Race was classified into three groups: White, Black, and Others (Asian or Pacific Islander, American Indian/Alaska Native). Marital status was divided into three groups: Married, single, and divorced (divorced, separated, and widowed). Histological grade was classified into four groups: Well‐differentiated, moderately differentiated, poorly differentiated, and undifferentiated. SEER stage was categorized into localized SEER stage, regional SEER stage, and distant SEER stage. Additionally, RPL histology was categorized as well‐differentiated (WDLS), myxoid (MLS), pleomorphic (PLS), dedifferentiated (DDLS), and other (round cell, mixed, angiomyoliposarcoma, fibroblastic and not otherwise specified) liposarcomas according to WHO classification.^1^ Patients with RPLs were assessed according to the sixth edition of the UICC TNM staging system. Surgery was classified into five groups: “No surgery”, “partial excision”, “total excision”, “radical surgery”, and “surgery, NOS”. The primary endpoints were overall survival (OS) and cancer‐specific survival (CSS). The former was calculated from time to death from any cause, while the latter was estimated from the time to death from RPLs.

**FIGURE 1:**
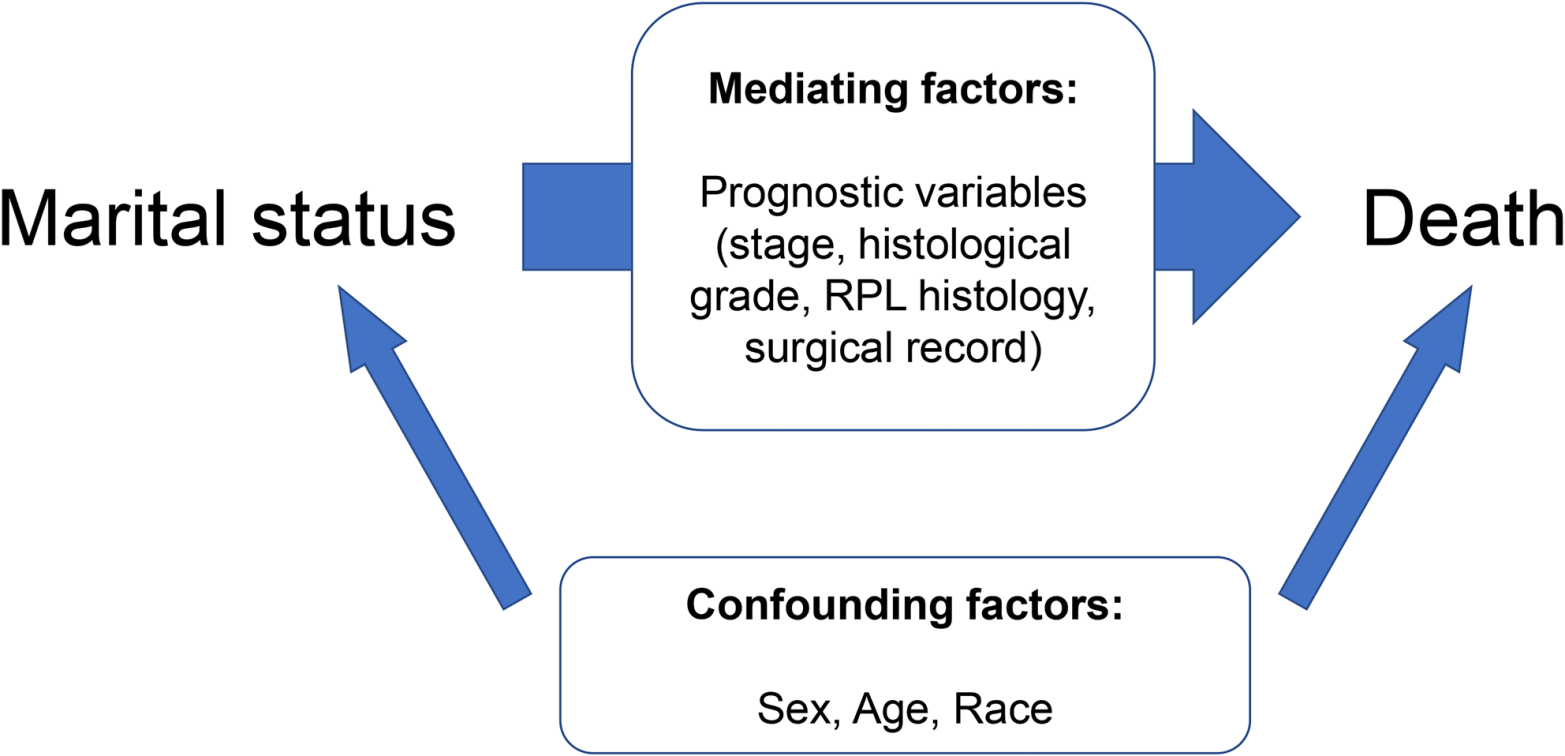
Confounding and mediating variables in the analytic model.

Covariates included basic characteristics (sex, age, and race) as confounding factors (Figure 1). In addition, factors known to be associated with RPL survival were included in the regression analyses as potential mediating variables that may explain marital disparities in survival (Figure 1),^31^, ^32^ namely, SEER stage (localized SEER stage, regional SEER stage, and distant SEER stage), histological grade (well‐differentiated, moderately differentiated, poorly differentiated, and undifferentiated), RPL histology (WDLS, MLS, PLS, and DDLS), and surgical record (complete resection, abdominal hemi‐evisceration, no surgery).

### 2.2 Statistical analysis

Baseline characteristics are expressed as frequencies. For categorical variables, Pearson's chi‐square test was used. In addition, propensity‐matched analysis was applied to reduce the effects of potential confounders on selection bias, and multiple logistic regression models were constructed to estimate the propensity score. In the models, marital status was the dependent variable and all baseline patient characteristics shown in Table 1 were evaluated as covariates. Propensity score matching was performed with a 1:1:1 matching protocol using a greedy matching approach with a caliper of 0.2.^33^ To assess pre‐match imbalance and post‐match balance, we estimated standardized differences for all the baseline covariates before and after matching.

**TABLE 1:** Characteristics of the included patients in SEER database before and after propensity score (PS) matching

	Before PS match									After PS match								
	Total *N* = 1211		Married *N* = 821 (67.80%)		Single *N* = 151 (12.47%)		Divorced *N* = 239 (19.73%)		*p* value^a^	Total *N* = 345		Married *N* = 115		Single *N* = 115		Divorced *N* = 115		*p* value^a^
	*N*	%	*N*	%	*N*	%	*N*	%		*N*	%	*N*	%	*N*	%	*N*	%	
Age																		
<65	648	53.51%	448	54.57%	112	74.17%	88	36.82%	<0.001	224	64.93%	76	66.09%	76	66.09%	72	62.61%	0.816
≥65	563	46.49%	373	45.43%	39	25.83%	151	63.18%		121	35.07%	39	33.91%	39	33.91%	43	37.39%	
Sex																		
Female	544	44.92%	333	40.56%	65	43.05%	146	61.09%	<0.001	175	50.72%	59	51.30%	60	52.17%	56	48.70%	0.860
Male	667	55.08%	488	59.44%	86	56.95%	93	38.91%		170	49.28%	56	48.70%	55	47.83%	59	51.30%	
Race																		
White	1018	84.06%	701	85.38%	115	76.16%	202	84.52%	0.002	286	82.90%	98	85.22%	92	80.00%	96	83.48%	0.475
Black	69	5.70%	35	4.26%	19	12.58%	15	6.28%		27	7.83%	5	4.35%	12	10.43%	11	9.57%	
Other^b^	124	10.24%	85	10.35%	17	11.26%	22	9.21%		32	9.28%	12	10.43%	11	9.57%	9	7.83%	
Histological grade^c^																		
I/II	718	59.29%	483	58.83%	101	66.89%	134	56.07%	0.095	213	61.74%	73	63.48%	73	63.48%	67	58.26%	0.643
III/IV	493	40.71%	338	41.17%	50	33.11%	105	43.93%		132	38.26%	42	36.52%	42	36.52%	48	41.74%	
Classification																		
WDLS	436	36.00%	303	36.91%	64	42.38%	69	28.87%	0.123	131	37.97%	48	41.74%	43	37.39%	40	34.78%	0.838
MLS	59	4.87%	34	4.14%	11	7.28%	14	5.86%		18	5.22%	3	2.61%	7	6.09%	8	6.96%	
PLS	27	2.23%	20	2.44%	2	1.32%	5	2.09%		7	2.03%	2	1.74%	2	1.74%	3	2.61%	
DDLS	479	39.55%	322	39.22%	53	35.10%	104	43.51%		135	39.13%	42	36.52%	45	39.13%	48	41.74%	
Other^d^	210	17.34%	142	17.30%	21	13.91%	47	19.67%		54	15.65%	20	17.39%	18	15.65%	16	13.91%	
SEER stage																		
Localized	594	49.05%	393	47.87%	78	51.66%	123	51.46%	0.793	183	53.04%	61	53.04%	59	51.30%	63	54.78%	0.831
Regional	505	41.70%	352	42.87%	60	39.74%	93	38.91%		132	38.26%	46	40.00%	46	40.00%	40	34.78%	
Distant	112	9.25%	76	9.26%	13	8.61%	23	9.62%		30	8.70%	8	6.96%	10	8.70%	12	10.43%	
Surgery																		
No surgery	80	6.61%	45	5.48%	10	6.62%	25	10.46%	0.026	19	5.51%	5	4.35%	7	6.09%	7	6.09%	0.356
Partial excision	436	36.00%	310	37.76%	54	35.76%	72	30.13%		110	31.88%	39	33.91%	41	35.65%	30	26.09%	
Total excision	180	14.86%	129	15.71%	15	9.93%	36	15.06%		39	11.30%	11	9.57%	9	7.83%	19	16.52%	
Radical surgery	515	42.53%	337	41.05%	72	47.68%	106	44.35%		177	51.30%	60	52.17%	58	50.43%	59	51.30%	

Abbreviations: DDLS, dedifferentiated liposarcoma; MLS, myxoid liposarcoma; PLS, pleomorphic liposarcoma; WDLS, well‐differentiated liposarcoma.

^a^ Categorical variables were assessed by the chi‐ squared test.

^b^ American Indian/AK Native, Asian/Pacific Islander, unknown.

^c^ Grade I: well differentiated; Grade II: moderately differentiated; Grade III: poorly differentiated; Grade IV: undifferentiated.

^d^ Round cell liposarcoma, mixed liposarcoma, angiomyoliposarcoma, fibroblastic liposarcoma and not otherwise specified liposarcoma.

In the matched cohort, the OS of marital status was drawn using the Kaplan–Meier method and assessed using the log‐rank test. Independent prognostic factors in RPLs were distinguished using univariate and multivariate Cox proportional hazard regression models, and the effects were expressed as hazard ratios (HRs) with 95% confidence intervals (CIs). Subsequently, we considered death due to the diagnosed cancer as the event of interest and death due to other causes as the censoring event. The Fine and Gray's competing risk analysis^34^ was performed to assess the cumulative incidence function (CIF) to explore every single variable incidence of each competing event. The proportional sub‐distribution hazard model was used to measure the effect of each prognostic variable on CSS. The significant prognostic variables (*p* < 0.05) in the univariate analyses were selected for the multivariate analysis. Multivariate analysis was proceeded using the stepwise regression model with backward elimination to identify independent risk factors. We used examining scaled Schoenfeld residuals^35^ to check the proportional hazards assumptions and did not observe violation of the proportional hazards assumptions. Similarly, we evaluated the effect of marital status stratified by age and sex on the prognostic outcome of patients with RPLs using multivariate analysis to analyze differences in survival by marital status. Interaction tests were performed using a Cox proportional hazards model. In addition, we further conducted sensitivity analyses to evaluate the association of potential unmeasured confounding by calculating the E value.^36^ Statistical analyses were performed using SPSS 20.0 (SPSS, Inc., Chicago, IL) and R software (version 4.1.2). All statistical tests were two tailed and used a significance level of 5%.

## 3 RESULTS

### 3.1 Patient baseline characteristics

**FIGURE 2:**
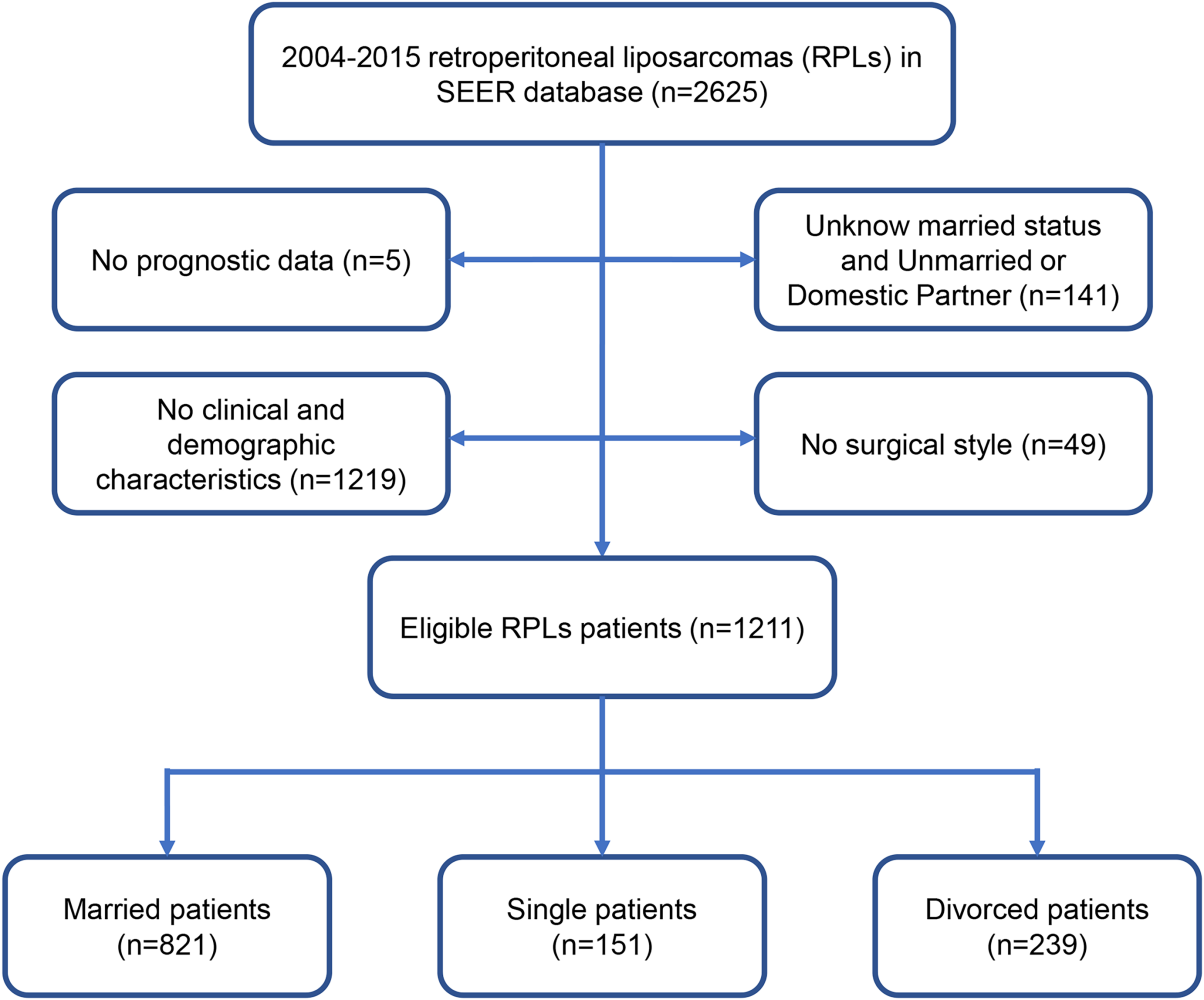
Flow chart of patient selection.

A total of 1211 eligible patients diagnosed with RPLs between 2004 and 2015 were identified in the SEER database. Afterwards, subjects were categorized into a married group (*n* = 821, 67.80%), a single group (*n* = 151, 12.47%) and a divorced group (n = 239, 19.73%), with the specific screening process shown in Figure 2. In the SEER database, 67.80% of patients were married. Men, who constituted 55.08% of the population, had the highest proportion (59.44%) of married patients and the lowest proportion (38.91%) of divorced patients. In addition, there were more younger patients (<65 years) among single patients than among married patients (74.17% vs. 54.57%). A younger age was least common (36.82%) among divorced patients. The overwhelming majority of the patients were white (84.06%), irrespective of marital status. Proportionally, no surgery was the most frequent surgery status for divorced patients (10.46%) and the least frequent status for married patients (5.48%). Additionally, there was no clear distinction between the different marital statuses in terms of histological grade, classification, SEER stage, AJCC stage, AJCC T stage, AJCC N stage, or AJCC M stage. Using the propensity score matching method, 151 patients from each group were matched. The groups were similar with regard to all the baseline variables after matching. Table 1 details the baseline characteristics according to the marital status of the patients with RPLs before and after propensity score matching.

### 3.2 Effects of marital status on OS and CSS

**FIGURE 3:**
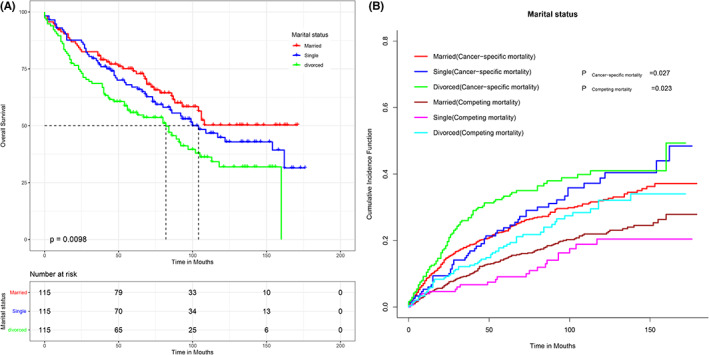
Overall survival (OS) and cancer‐specific survival (CSS) of patients. The Kaplan–Meier survival curves of OS (A) and the cumulative incidence function curves of CSS (B) by marital status.

Figure 3 depicts the Kaplan–Meier survival curves of OS and the cumulative incidence function curves of CSS according to marital status. The results showed that married patients had significantly longer OS and CSS than divorced, separated, or widowed patients (OS: *p* = 0.003 and CSS: *p* = 0.003). However, the Kaplan–Meier survival curves and the cumulative incidence function curves revealed no significant difference in CSS and OS between married and single patients (OS: *p* = 0.260 and CSS: *p* = 0.116). To assess RPL prognosis‐related factors, significant prognostic variables (*p* < 0.05) identified in univariate analyses were selected for inclusion in the multivariate analysis for further exploration of their covariate‐adjusted effects on survival time. After multivariate adjustment, marital status, age, sex, histological grade, classification, SEER stage, and surgical options were identified as independent prognostic factors for OS and CSS in patients with RPLs. The results are summarized in Tables 2 and 3.

**TABLE 2:** Univariate analysis for overall survival and cancer‐specific survival

Cetegory	*N*	Overall survival *p* (Log‐rank test)	Cancer‐specific survival *p* (Fine and Gray's test)
Age			
<65	224	Ref.	Ref.
≥65	121	<0.001	<0.001
Sex			
Female	175	Ref.	Ref.
Male	170	<0.001	0.006
Race			
White	286	Ref.	Ref.
Black	27	0.272	0.686
Other^a^	32	0.520	0.711
Histological grade^b^			
I/II	213	Ref.	Ref.
III/IV	132	<0.001	<0.001
Classification			
WDLS	131	Ref.	Ref.
MLS	18	0.003	<0.001
PLS	7	<0.001	<0.001
DDLS	135	<0.001	<0.001
Other^c^	54	<0.001	<0.001
SEER stage			
Localized	183	Ref.	Ref.
Regional	132	<0.001	<0.001
Distant	30	<0.001	<0.001
AJCC stage			
I/II	206	Ref.	Ref.
III/IV	139	<0.001	<0.001
AJCC T stage			
T1	11	Ref.	Ref.
T2	334	0.903	0.196
AJCC N stage			
N0	336	Ref.	Ref.
N1	9	<0.001	<0.001
AJCC M stage			
M0	326	Ref.	Ref.
M1	19	<0.001	<0.001
Surgery			
No surgery	19	Ref.	Ref.
Partial excision	110	<0.001	<0.001
Total excision	39	<0.001	<0.001
Radical surgery	177	<0.001	<0.001
Marital status			
Married	115	Ref.	Ref.
Single	115	0.260	0.116
Divorced	115	0.003	0.003

*Note*: The *p* value in the column of univariate analysis means that the variable was selected in the next multivariate analysis.

Abbreviations: DDLS, dedifferentiated liposarcoma; MLS, myxoid liposarcoma; PLS, pleomorphic liposarcoma; Ref., referent; RPLs, retroperitoneal liposarcomas; WDLS, well‐differentiated liposarcoma.

^a^ American Indian/AK Native, Asian/Pacific Islander, unknown.

^b^ Grade I: well differentiated; Grade II: moderately differentiated; Grade III: poorly differentiated; Grade IV: undifferentiated.

^c^ Round cell liposarcoma, mixed liposarcoma, angiomyoliposarcoma, fibroblastic liposarcoma and not otherwise specified liposarcoma.

**TABLE 3:** Multivariate analysis for overall survival and cancer‐specific survival

Cetegory	Overall survival	*p*	Cancer‐specific survival	*p*
	HR (95% CI)		HR (95% CI)	
Age				
<65	Ref.	Ref.	Ref.	Ref.
≥65	1.68 (1.42, 1.97)	<0.001	1.28 (1.04, 1.58)	0.019
Sex				
Female	Ref.	Ref.	Ref.	Ref.
Male	1.29 (1.09, 1.53)	0.003	1.16 (0.94, 1.43)	0.180
Histological grade^a^				
I/II	Ref.	Ref.	Ref.	Ref.
III/IV	1.85 (1.45, 2.36)	<0.001	2.26 (1.64, 3.11)	<0.001
Classification				
WDLS	Ref.	Ref.	Ref.	Ref.
MLS	1.54 (1.04, 2.28)	0.033	2.08 (1.27, 3.4)	0.004
PLS	1.40 (0.81, 2.43)	0.233	1.98 (1.02, 3.86)	0.044
DDLS	1.73 (1.29, 2.3)	<0.001	2.18 (1.47, 3.24)	<0.001
Other^b^	1.43 (1.1, 1.86)	0.007	1.46 (1.00, 2.13)	0.050
SEER stage				
Localized	Ref.	Ref.	Ref.	Ref.
Regional	1.38 (1.16, 1.65)	<0.001	1.52 (1.20, 1.92)	<0.001
Distant	2.02 (1.55, 2.62)	<0.001	2.87 (2.11, 3.90)	<0.001
AJCC stage				
I/II	Ref.	Ref.	Ref.	Ref.
III/IV	1.51 (0.62, 3.68)	0.361	1.28 (0.74, 2.19)	0.364
AJCC N stage				
N0	Ref.	Ref.	Ref.	Ref.
N1	0.88 (0.55, 1.40)	0.592	0.78 (0.45, 1.34)	0.364
AJCC M stage				
M0	Ref.	Ref.	Ref.	Ref.
M1	1.52 (0.93, 2.47)	0.093	1.30 (0.76, 2.22)	0.341
Surgery				
No surgery	Ref.	Ref.	Ref.	Ref.
Partial excision	0.27 (0.2, 0.36)	<0.001	0.23 (0.16, 0.33)	<0.001
Total excision	0.30 (0.22, 0.42)	<0.001	0.28 (0.19, 0.42)	<0.001
Radical surgery	0.24 (0.18, 0.32)	<0.001	0.21 (0.15, 0.29)	<0.001
Marital status				
Married	Ref.	Ref.	Ref.	Ref.
Single	1.21 (0.81, 1.81)	0.357	1.36 (0.80, 2.29)	0.252
Divorced	1.66 (1.12, 2.46)	0.012	1.90 (1.13, 3.19)	0.015

Abbreviations: CI, confidence interval; DDLS, dedifferentiated liposarcoma; HR, hazard ratio; MLS, myxoid liposarcoma; PLS, pleomorphic liposarcoma; Ref., referent; RPLs, retroperitoneal liposarcomas; WDLS, well‐differentiated liposarcoma.

^a^ Grade I: well differentiated; Grade II: moderately differentiated; Grade III: poorly differentiated; Grade IV: undifferentiated.

^b^ Round cell liposarcoma, mixed liposarcoma, angiomyoliposarcoma, fibroblastic liposarcoma and not otherwise specified liposarcoma.

Divorced patients had worse survival outcomes than married patients (OS: HR = 1.66 (95% CI, 1.12, 2.46)); CSS: HR = 1.90 (95% CI, 1.13, 3.19)). The E‐values calculated for this effect in our sensitivity analysis were 2.19 for OS and 2.49 for CSS, and for the upper bound of the 95% CI, these values were 1.38 for OS and 1.40 for CSS. These results suggest no substantial unmeasured confounding. Single patients did not significantly differ from married patients in terms of OS (HR = 1.21 [95% CI, 0.81, 1.81]) or CSS (HR = 1.36 [95% CI, 0.80, 2.29]). We also calculated the E‐value for this effect in our sensitivity analysis and obtained values of 1.54 for OS and 1.78 for CSS, and for the upper bound of the 95% CI, these values were 1.00 for OS and 1.00 for CSS. These results also suggest no substantial unmeasured confounding. The results are summarized in Table S1.

### 3.3 Effects of marital status on OS and CSS according to age stratification

As shown in Table 4, divorced patients experienced shorter OS than married patients in each age group (patients < 65 years of age: HR = 1.44 (95% CI, 1.04, 1.99); patients ≥ 65 years of age: HR = 1.44 (95% CI, 1.04, 1.99)). However, the differences in CSS between divorced and married patients, which were not statistically significant in patients ≥ 65 years of age (HR = 1.46 [95% CI, 1.07, 2.00]), were statistically significant in patients < 65 years of age (HR = 1.33 [95% CI, 0.89, 1.98]). Additionally, there were no significant differences in CSS and OS between married and single patients (patients < 65 years of age: (OS: HR = 1.10 (95% CI, 0.80, 1.51); CSS: HR = 1.15 (95% CI, 0.79, 1.67)); patients ≥ 65 years of age: OS: HR = 1.02 (95% CI, 0.67, 1.54); CSS: HR = 1.19 (95% CI, 0.70, 2.04). The abovementioned results revealed that marital status influenced survival in patients with RPLs at each age, but no statistically significant interaction was observed (Figure S1). However, marital status had a stronger influence on older patients than it did on younger patients, notably for CSS. These results demonstrate that although marital status influenced survival in patients with RPLs at each age, divorce played a significant detrimental role in mortality in patients older than 65 years.

**TABLE 4:** Hazard ratios for the association between marital status and survival by age and sex

Cetegory	Single (vs. Married)				Divorced (vs. Married)			
	Overall survival		Cancer‐specific survival		Overall survival		Cancer‐specific survival	
	HR (95% CI)	*p*	HR (95% CI)	*p*	HR (95% CI)	*p*	HR (95% CI)	*p*
Age								
<65	1.10 (0.80, 1.51)	0.553	1.15 (0.79, 1.67)	0.462	1.44 (1.04, 1.99)	0.029	1.33 (0.89, 1.98)	0.162
≥65	1.02 (0.67, 1.54)	0.938	1.19 (0.70, 2.04)	0.522	1.36 (1.08, 1.72)	0.010	1.46 (1.07, 2.00)	0.018
Sex								
Female	0.95 (0.63, 1.43)	0.792	1.31 (0.81, 2.09)	0.269	1.81 (1.38, 2.39)	<0.001	1.93 (1.36, 2.73)	<0.001
Male	0.99 (0.72, 1.35)	0.932	1.01 (0.68, 1.50)	0.952	1.47 (1.12, 1.92)	0.005	1.29 (0.90, 1.86)	0.172

Abbreviations: CI, confidence interval; HR, hazard ratio.

### 3.4 Effects of marital status on OS and CSS according to sex stratification

As shown in Table 4, divorced patients of both sexes had shorter OS times than married patients (female patients: HR = 1.81 (95% CI, 1.38, 2.39); male patients: HR = 1.47 (95% CI, 1.12, 1.92)). However, the differences in CSS between divorced and married patients, which were not statistically significant for male patients (HR = 1.29 [95% CI, 0.90, 1.86]), were statistically significant for female patients (HR = 1.93 [95% CI, 1.36, 2.73]). Additionally, there were no significant differences in CSS and OS between married and single patients (female patients: (OS: HR = 0.95 (95% CI, 0.63, 1.43); CSS: HR = 1.31 (95% CI, 0.81, 2.09)); male patients: (OS: HR = 0.99 (95% CI, 0.72, 1.35); CSS: HR = 1.01 (95% CI, 0.68, 1.50)). The abovementioned results indicated that marital status influenced survival in patients with RPLs in female and male patients, but no statistically significant interaction was observed (Figure S1). However, marital status had a stronger influence on female patients than on male patients, notably for CSS. These results demonstrate that although marital status influenced survival in patients with RPLs in female and male patients, divorce had a greater impact on prognosis in females than males.

## 4 DISCUSSION

Retroperitoneal soft tissue sarcomas (RPSs), a heterogeneous group of rare malignancies, comprise 0.15% of all malignancies and approximately 15% of all soft tissue sarcomas.^32^, ^37^ RPLs are the most common pathological type, and their early diagnosis and efficient treatment represent considerable challenges due to the size and deep location of RPSs. As a consequence, recurrence the following surgery occurs in the majority of patients with RPLs and is the cause of death in most patients.^38^ In previous studies, a large number of clinical and histopathological features have been widely used to predict the prognosis of RPLs and guide clinical treatment strategies.^15^, ^16^, ^17^, ^18^, ^19^, ^20^ Moreover, with the increased understanding of human health and the consequent advancements in healthcare, socio‐psychosocial factors, including marital status, are gaining increasing attention as a key player influencing the prognosis of cancer.^21^, ^27^ Several studies have shown that the prognosis of married patients is significantly better than that of unmarried patients for several types of cancer, including breast cancer, thyroid cancer, multiple myeloma, bladder cancer, and prostate cancer.^39^, ^40^, ^41^, ^42^, ^43^, ^44^ However, few studies on marital status among RPL patients have been reported. In this study, we investigated the association between marital status and survival outcomes in a large cohort of patients with RPLs. Given that age and sex are important prognostic factors for RPL, our study evaluated the prognostic impact of marital status on patients with RPL in different age groups and sex groups. To our knowledge, this is the first demonstration of the interaction of age/sex and marital status in RPL survival.

Our findings suggested that divorced patients had more advanced disease stages at diagnosis, a finding that was also shown in patients with mycosis fungoides, lung neuroendocrine neoplasms, and melanoma.^45^, ^46^, ^47^ There are several potential interpretations of this phenomenon. Physical examination of married patients is conducted regularly under the supervision of their spouses before being diagnosed, and married patients are more likely to be diagnosed at an earlier stage. For unmarried patients, early‐stage lesions might be overlooked by the patients themselves. Moreover, cancer patients have high levels of anxiety, depression, and distress. Previous studies have revealed that psychological stress could lead to tumor progression by negatively affecting the normal function of the immune and endocrine systems.^45^, ^48^, ^49^ Married patients can share their emotional burden and receive emotional and financial support from their spouses.^40^ The univariate analysis in the present study revealed that married patients had significantly superior OS and CSS compared to divorced patients. To exclude the impact of other factors on survival prognosis, we conducted a multivariate analysis. The results revealed that marital status is an independent prognostic factor for OS and CSS, demonstrating that marital status may influence survival outcomes independent of other factors. Only 5.48% of married patients and 6.62% of single patients were not treated with surgery, which is significantly lower than the 10.46% of divorced patients. It is well established that complete surgical resection is an effective method to improve survival rates for most patients with RPLs. Therefore, married patients may be more likely to benefit from surgical resection than divorced patients, which may be one of the reasons that married patients have a better prognosis than divorced patients. As noted above, married patients have greater attention, better care, and social support, which demonstrates a positive impact on the survival of RPL patients. Interestingly, we found that there was no significant difference between single and married patients. In addition, the study revealed that there were fewer younger patients among divorced patients (<65 years) than among married patients (36.82% vs. 54.57%) and that a young age was most common among single patients (74.17%). Using the propensity score matching method, 151 patients from each group were matched. The groups were similar with regard to all the baseline variables after matching.

In the matched cohort, the results showed that marital status was identified as an independent prognostic factor for survival in patients with RPLs and that married patients had significantly longer OS and CSS than divorced, separated, or widowed patients. In addition, we evaluated the effect of marital status stratified by age and sex on the prognostic outcome of patients with RPLs to analyze differences in survival by marital status. Our results demonstrated that marriage has a significant protective effect on older (≥65 years) and female patients.

Despite these findings regarding marital status and survival, some limitations of this study are important to note. First, our study has the intrinsic limitations and biases of any retrospective analysis, limiting our ability to explore causal relationships between marital status and survival. Second, since only the marital status of the patients at diagnosis is recorded in SEER, we could not assess the changes in marital status after diagnosis. These changes might affect the final survival outcomes. Third, the SEER database did not record the quality of marriage, which also may lead to discrepant survival outcomes. Fourth, some information relating to both marital status and prognosis of patients with RPLs, such as reproductive history and levels of hormones, was unavailable in the SEER database. Fifth, information about socioeconomic status and education was not contained in the database. Due to these limitations, the findings reported in this article need to be interpreted with caution.

In summary, the results of our study underscore the importance of marital status as a prognostic factor for RPLs. Divorced, separated or widowed marital status was an independent prognostic factor for worse CSS and OS outcomes. This effect was especially pronounced in older people and females. Hence, marital status is worthy of attention when doctors communicate with patients who are diagnosed with RPLs. Moreover, psychological support should be considered for patients without a partner. Further prospective studies are necessary to further elucidate the impact of marital status on the prognosis of patients with RPLs.

## AUTHOR CONTRIBUTIONS

Yiding Liand Guiling Wuparticipated in the design of this study and wrote the manuscript. Liu Hongand Daiming Fanconceived the original idea, supervised the overall direction and planning of the project. Yujie Zhang, Wanli Yang, Xiaoqian Wang, Lili Duan, Liaoran Niu, Junfeng Chen, Wei Zhou, and Jinqiang Liucontributed to the acquisition of the data, analysis, and interpretation of the data. All authors contributed to the article and ap‐proved the submitted version.

## CONFLICT OF INTEREST

The authors declare that they have no competing interests.

## ETHICAL APPROVAL STATEMENT

Research Data Agreement Form was required by the National Cancer Institute's SEER Program prior to access to the de‐identified SEER dataset. Since the de‐identified data were used, approval from the Ethics Committee of the Xijing Hospital and patients' informed consent were not required.

## Supporting information


Figure S1

Table S1

## Data Availability

All data used in this paper are accessible from Surveillance, Epidemiology, and End Results (SEER) database after a reasonable submission of a request for access to the data at https://seer.cancer.gov/.
