# Supplementary material for: Effects of marital status on survival of retroperitoneal liposarcomas stratified by age and sex: A population‐based study
Source: Cancer Med. 2022 Jun 27;12(2):1779–90. doi: 10.1002/cam4.4962 (PMC9883417; doi:10.1002/cam4.4962)
Supplement: Supplementary file 1 — Figure S1 [file CAM4-12-1779-s002.docx]

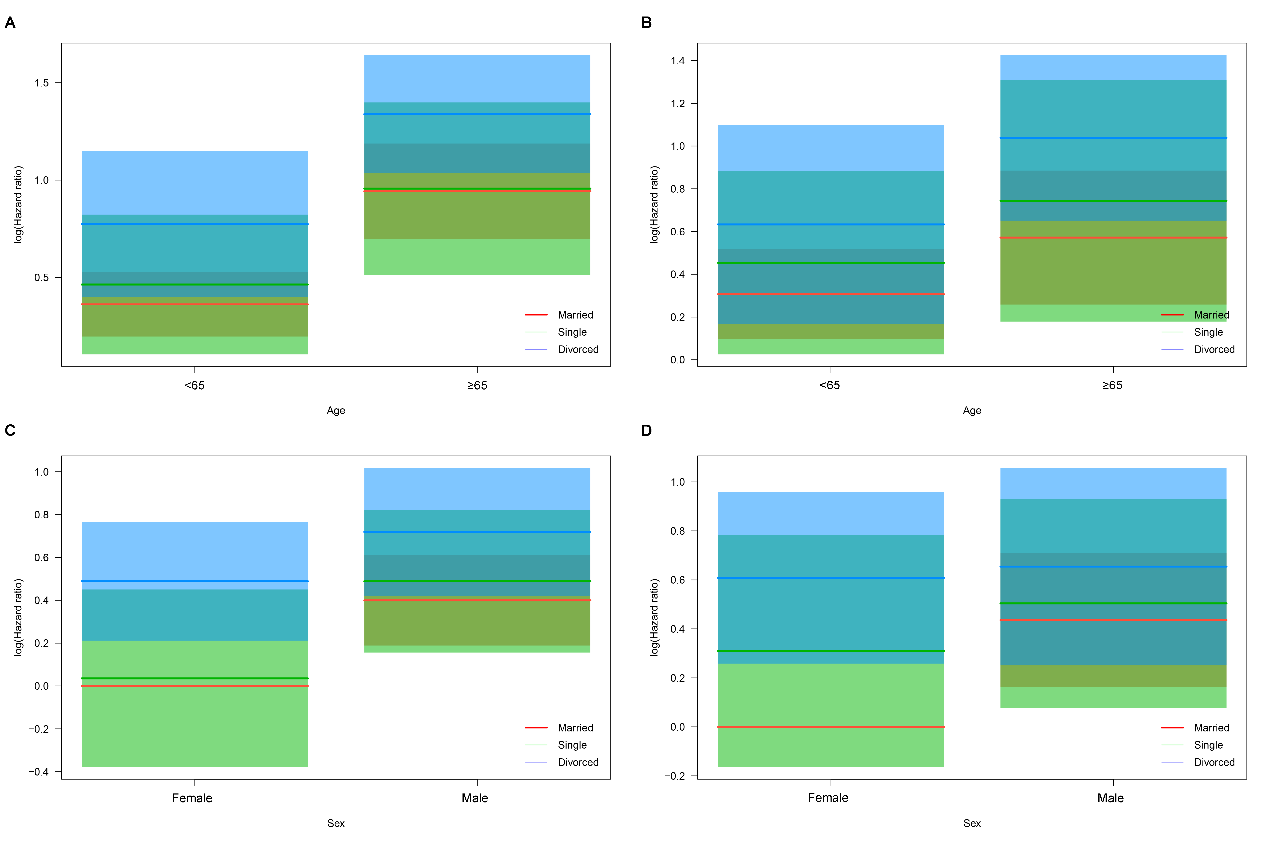


**Figure S1** Visualizations of the interaction effects using Cox regression of age/sex and marital status. (A) the interaction effects between age and marital status in OS; (B) the interaction effects between age and marital status in CSS; (C) the interaction effects between sex and marital status in OS; (D) the interaction effects between sex and marital status in CSS.
